# Adaptation of a standard extended-release naltrexone (XR-NTX) protocol for rural re-entering offenders with OUD

**DOI:** 10.1186/s40352-021-00130-0

**Published:** 2021-02-05

**Authors:** Michele Staton, Hannah K. Knudsen, Sharon L. Walsh, Carrie Oser, Erika Pike, Michelle Lofwall

**Affiliations:** 1 Department of Behavioral Science, University of Kentucky College of Medicine, Medical Behavioral Science Building, Lexington, KY 40536-0086 USA; 2 Center on Drug and Alcohol Research, University of Kentucky, 845 Angliana Ave, Lexington, KY 40508 USA; 3 Department of Sociology, University of Kentucky College of Arts and Sciences, Lexington, KY 40506-0027 USA

**Keywords:** XR-NTX, Rural offenders, OUD, Community supervision

## Abstract

**Background:**

Despite a growing body of empirical support for the effectiveness of extended-release naltrexone (XR-NTX) to reduce opioid relapse among people with opioid use disorder (OUD) transitioning from a correctional facility to the community, continuity of care following release remains challenging. This paper describes a research-based adaptation of a state’s standard of care XR-NTX protocol using the ADAPT-ITT framework for delivery in a non-traditional, non-treatment, community criminal justice setting (P&P office), as well as the expansion of services by a local Federally Qualified Health Center (FQHC) provider who would, for the first time, be going to the jail and P&P office to provide XR-NTX and related treatment.

**Method:**

The present study focuses on the first seven phases (Assessment through Training) of the ADAPT-ITT framework in the adaptation of the Department of Corrections (DOC) protocol in preparation for a pilot trial for induction in a rural jail and during the transition to a rural community. Expert clinical review and focus groups with key stakeholders in criminal justice supervision and the local providers in the FQHC informed the needed adaptations to the existing XR-NTX protocol for initiation at the jail and ongoing administrations in the community.

**Results:**

Findings from stakeholder focus groups, study team review, topical expert review, and a theater test suggested that there were critical adaptations needed in both content and context at the patient and clinic level.

**Conclusion:**

Health and justice officials should consider the need to tailor and adapt evidence-based approaches for real-world locations that high-risk, justice-involved individuals visit in order to reduce barriers and increase access to critically needed treatment for OUD.

## Background

While the opioid epidemic continues to pose a tremendous burden in a number of states, Kentucky has experienced particularly devastating public health and public safety consequences associated with the non-medical use of prescription opioids and the insurgence of heroin and illicit fentanyl (e.g., Faryar et al., 2017; Slavova, Rock, Bush, Quesinberry, & Walsh, 2020; Victor, Walker, Cole, & Logan, 2017). Despite the empirical support for efficacy of Federal Drug Administration (FDA)-approved medications to treat opioid use disorder ([MOUD]; Lee et al., 2015; Perry et al., 2013; Sharma et al., 2016; Syed & Keating, 2013), only about 1 in 5 individuals with OUD nationally receive any form of treatment and fewer received medication (Saloner & Karthikeyan, 2015). Even fewer receive treatment in resource-deprived areas, such as Appalachian Kentucky, due to the limitations of available services and stigma associated with seeking treatment (e.g., Oser & Harp, 2015).

Because an estimated 80% of individuals in the criminal justice system have a history of substance use and about 20% report regular non-medical opioid use specifically (Bronson, Stroop, Zimmer, & Berzofsky, 2017), incarceration can serve as a critical window of opportunity to initiate OUD treatment for affected individuals. However, the use of MOUD remains widely underutilized in corrections. National surveys indicate that about half of state jails and prisons use some form of MOUD, but often limit this to pregnant women or management of withdrawal (Friedmann et al., 2012; Grella, Ostile, Scott, Dennis, & Carnavale, 2020; Oser, Knudsen, Staton-Tindall, Taxman, & Leukefeld, 2009). The use of MOUD is often more restricted for individuals on community supervision (i.e., probation and parole [P&P]), with some P&P districts prohibiting the use of MOUD (Friedmann et al., 2012). In addition, P&P officers typically have limited training on the potential benefits of MOUD (Mitchell et al., 2016). These challenges within the justice system may contribute to limited treatment opportunities for individuals as they transition from correctional settings to the community, particularly individuals re-entering rural areas from jail who face limited access to health and behavioral health care in general and limited availability of MOUD, more specifically. These challenges are often exacerbated by lack of insurance coverage during the transition to the community, which may also serve as a significant barrier to OUD treatment linkages, enrollment, and retention (Justesen et al., 2020).

Extended-release naltrexone (XR-NTX) reduces the likelihood of opioid relapse among offenders re-entering the community (Friedmann, Wilson, Hoskinson, Poshkus, & Clarke, 2018; Gordon et al., 2017; Lee et al., 2016). As a sustained-release opioid antagonist, the primary advantage is that it *should* reduce challenges associated with adherence by providing 30 days of medication, which can be advantageous during the community re-entry period following release (ASAM, 2015; Lee et al., 2016). It also has no abuse liability, special regulations, or provider training requirements. In a proof-of-concept trial, Lee et al. (2015) found that XR-NTX acceptance was high among males with OUD from New York City jails with 88% initiating medication prior to prison release; XR-NTX patients were significantly less likely to return to opioid use after release. Medication adherence during community re-entry is critical, however, in understanding outcomes. One study demonstrated that individuals transitioning from urban area prisons in Baltimore were more likely to remain opioid abstinent if they received six monthly XR-NTX administrations in the community post-release compared to those who received fewer community administrations (Gordon et al., 2015). However, another study showed no differences between XR-NTX alone and XR-NTX in combination with patient navigation on opioid outcomes, largely attributed to low treatment adherence in the community and high rates of re-incarceration at follow-up (Farabee, Condon, Hallgren, & McCrady, 2020). The community re-entry period following release can be stressful due to housing, income, transportation, and relationship problems. Additionally, adherence to a medication regime that requires developing a relationship with a new health care provider with at least monthly clinic visits can also be challenging (Velasquez et al., 2019).

The current study builds on the empirical evidence for positive outcomes associated with XR-NTX for re-entering offenders, but expands the work to consider an innovative community model for ongoing treatment following jail release. In March 2015, state legislators passed Senate Bill 192 (SB192), a comprehensive drug policy that increased funding for MOUD (specifically XR-NTX) in Kentucky prisons and jails (Dantzler, 2015). The state Department of Corrections (DOC) Division of Addiction Services worked closely with a national pharmaceutical company and correctional health care officials to develop a clinical protocol for screening and administration of XR-NTX. Eligibility requirements included: an OUD diagnosis, within 60 days of anticipated release, a negative urine drug screen before a 3-day oral 50 mg/day naltrexone challenge, and not being pregnant. The DOC protocol included two XR-NTX administrations 1 month apart during incarceration with a referral to a community case worker (social service clinician) for coordination of and linkage to continued XR-NTX treatment and recovery services following release. The DOC protocol was, however, limited to offenders who received treatment in state Substance Abuse Programs (SAP), which are modified therapeutic communities mostly centralized in state prisons. Kentucky has 76 jails across 120 counties that house nearly 24,500 inmates daily (Kentucky DOC, 2018); only 22 of these jails (29%) have SAP programs with about 1600 treatment slots annually. Thus, there was a significant need to adapt the DOC protocol for XR-NTX administration to increase access to the medication in a rural jail setting and to reduce barriers to continued XR-NTX treatment upon community re-entry. The research-based adaptation, supported by a federal research grant, is the focus of this paper.

Because adherence to XR-NTX can be challenging post-release, adapting clinical XR-NTX continuity of care protocols for delivery in settings where justice-involved individuals are likely to visit, such as the community P&P office, has the potential to reduce burdens following jail release and improve MOUD adherence. The co-location of XR-NTX treatment in P&P offices where individuals are expected to visit during re-entry has not been examined, and thus requires significant adaptations to the existing state DOC XR-NTX standard of care protocol (which is referred to as ‘DOC protocol’ hereafter). Furthermore, being attentive to the resources and constraints of the rural Appalachian context where the intervention is to be implemented is critically important.

To systematically adapt the DOC protocol and fit the needs of a population (individuals with OUD incarcerated in jail) (Rabin, Brownson, Haire-Joshu, Kreuter, & Weaver, 2008), this study is guided by the ADAPT-ITT framework. The ADAPT-ITT framework was originally developed to guide the systematic adaptation of evidence-based interventions (EBIs) targeting HIV risk through eight sequential phases - **A**ssessment, **D**ecision, **A**dministration, **P**roduction, **T**opical experts, **I**ntegration, **T**raining, and **T**esting (Wingood & DiClemente, 2008). Recent research using the ADAPT-ITT framework has emphasized its utility with HIV risk reduction behavioral interventions (e.g., Khumsaen & Stephenson, 2017; Latham et al., 2010; Sullivan et al., 2014); however, this is the first study to utilize the ADAPT-ITT framework for a medication administration protocol. The ADAPT-ITT framework is well justified for this purpose because it draws heavily on stakeholder feedback to better understand needed adaptions to both content of the protocol, as well as context in the jail and in the rural community. It also provides a systematic framework for building upon the assessment of needed adaptation, making decisions about those adaptations, and evaluating those changes in an iterative way prior to launching a pilot trial. This paper describes the research-based adaptation of the DOC protocol using the ADAPT-ITT framework for delivery in a non-traditional, non-treatment, community criminal justice setting (P&P office), as well as the expansion of services by a local FQHC provider who would, for the first time, be going to the jail and P&P office to deliver comprehensive XR-NTX care.

## Method

The present study focuses on the first seven phases (Assessment through Training) of the ADAPT-ITT framework in the adaptation of the DOC protocol in preparation for a pilot trial in a rural jail and P&P office as part of a federally-supported research study. With implementation of the Assessment phase in February, 2019, the timeline for completion of all protocol adaptation phases was approximately 7 months, with pilot trial testing beginning in Septembe 2019. This study was reviewed and approved by the university Institutional Review Board and registered on ClinicalTrials.gov (NCT03447743).

### Study setting

The community setting for this study is one rural county located in central Appalachia with a population of nearly 29,000. The county was selected because it is one of the hardest hit areas by the opioid epidemic, and it is one of the Appalachian counties identified as economically distressed with economic rankings in the worst 10% of the counties in the nation (ARC, 2020). The county also includes strong community partners in the local jail, the P&P office, and a rural FQHC who enthusiastically committed support for the study. The local county jail has a daily census of approximately 150 and no current state-supported treatment (SAP program), but jail leadership were interested in providing treatment for individuals in custody. Based on previous studies, the majority of jail inmates have a history of substance use, particularly opioid use (Staton et al., 2018). The selected county also has a P&P field office, which organizationally falls under the leadership of the Kentucky DOC. The majority of individuals on community supervision also have a history of substance use-related offenses, and a common reason noted for parole/probation violations was return to opioid use (Kentucky DOC, 2018). The partnership for this project also includes a FQHC designated by the state of Kentucky as a Rural Health Clinic, which also is a KY DOC contracted site for community treatment. The FQHC is in the same county as the jail. Specifically, this FQHC administers XR-NTX (Vivitrol®) with counseling and case management services to anyone released from prison/jail who is interested in seeking OUD treatment.

### Assessment

This first phase of the ADAPT-ITT process focuses primarily on assessing the needs of the target population for the intervention including risks and logistical challenges with delivering the intervention (Wingood & DiClemente, 2008). Consistent with other studies using the ADAPT-ITT framework (Khumsaen & Stephenson, 2017; Munro-Kramer et al., 2020), focus groups were used in the current study to provide the formative work needed during the Assessment phase in order to assess the needs of the target population and adapt intervention content. Two focus groups were conducted with key stakeholder groups in March–April 2019, the first with Department of Corrections (DOC) staff including administrators, probation and parole officers, treatment staff, and re-entry staff (*n* = 9), and the second with XR-NTX health care providers and staff working at the FQHC who would provide the MOUD at the jail and post-release at either the P&P office or the FQHC (*n* = 5). Participation was offered to all DOC administrators and staff and all FQHC staff working with the target population. The procedures were approved by the university Institutional Review Board, participants provided written informed consent, and staff were reminded that participation in the groups was completely voluntary. Staff were not provided with a monetary incentive, but refreshments were provided during the groups. Both groups were conducted in private conference rooms, facilitated by the principal investigator (PI), and audio-recorded for transcription. The groups were guided by semi-structured interview guides developed by the research team intended to assess attitudes toward MOUD, available resources in the area, potential concerns about XR-NTX, perceptions of client issues, and feedback on possible assessment, evaluation, and medication administration procedures. Each group took approximately 1 hour. Transcripts from both focus groups were coded and qualitatively analyzed using content analysis. Content analysis included specific focus on identified needs of the target population (e.g., OUD diagnosis, medically eligible to initiate medication, re-entry challenges to engaging in treatment) and necessary changes to the DOC protocol to ensure continuity of care in the community.

### Decision

The Decision phase of the ADAPT-ITT process typically involves a review of the evidence for the EBI, deciding on the EBI for the target population, and making decisions about needed adaptations to the EBI for the proposed population (Wingood & DiClemente, 2008). The empirical literature on delivery of XR-NTX for justice-involved individuals in transition from jail to the community provided the foundation for the current study (e.g., Friedmann et al., 2018; Gordon, Kinlock, et al., 2017; Lee et al., 2016). In addition, documentation including the coded transcripts from the focus groups and content analysis were used to inform decisions about specific recommended adaptations to content, delivery strategies, and frequency/method of contact.

### Administration

The Administration phase of the ADAPT-ITT process involves a pilot test of the adapted intervention, typically referred to as a “theater test” (Wingood & DiClemente, 2008). The theater test involves a demonstration, or walk through, of the adapted intervention with individuals either from the target population or individuals who can simulate the needs and behaviors of the target population. The adapted version underwent a “theater test” of all study procedures in a private room in the jail (screening, medical evaluation, initial XR-NTX administration procedures) and in a private room in the P&P office (on-going community XR-NTX administration) with an experienced physician co-investigator (MRL) simulating the role of the patient. With more than 15 years of clinical and research experience with individuals with OUD, our volunteer realistically portrayed different scenarios for the research staff and FQHC clinical team while walking through each section of the adapted protocol. The research study coordinator and PI were present during the theater test to take notes on each section of the protocol for additional modifications.

### Production and topical expert review

The Production phase includes finalizing a working draft of the adapted protocol with a focus on maintaining the critical elements of XR-NTX delivery with modifications tailored to fit the needs of the client population (content) during the period of transition between jail to the community (context). This phase of ADAPT-ITT is closely followed by the Topical Expert review which includes review of the protocol by a designated consultant with expertise in the scientific area (Wingood & DiClemente, 2008). For the current study, notes from the theater test were incorporated into the adapted protocol and reviewed by the study team, including two co-investigators (MRL, SLW) with extensive clinical and research experience with MOUD. Feedback from the team was used to produce a more refined version of the XR-NTX protocol for review by a paid study consultant (Topical Expert). The Topical Expert was a physician researcher from an outside University with previous and on-going clinical and research trial expertise involving the delivery of XR-NTX during incarceration as individuals prepare for community re-entry.

### Integration

The Integration phase of ADAPT-ITT includes incorporating all of the suggested changes during the Topical Expert review into a more refined draft of the adapted protocol. Integration of all feedback should be done with an eye to maintaining the core components of the protocol and tailoring specifically to the target population (Wingood & DiClemente, 2008). For the current study, feedback from the topical expert and study team was then integrated into the final draft of the adapted XR-NTX protocol. The PI, physician Co-I, and study coordinator met with key stakeholders from the partnering jail and P&P office, as well as the FQHC clinical team to review the protocol and assess feasibility.

### Training

Training is the last ADAPT-ITT phase before pilot testing. This phase typically includes all study personnel including clinical and research staff (Wingood & DiClemente, 2008). One full-day interactive training session was conducted with research staff on the adapted XR-NTX protocol for the current study.

## Results

### Assessment

The first focus group, which included representatives from the DOC, indicated there was general agreement that, despite seeing an increase in non-medical use of buprenorphine in the area (including by injection), individuals were usually allowed to continue needed and prescribed MOUD while on community supervision. Regarding initiating medication prior to jail release and continuing medication during supervision, one of the significant challenges noted by the group was the feasibility of identifying potential participants. The local jail is a regional facility and many individuals housed there transition to supervision in surrounding counties, making continuing care with the local FQHC infeasible. The group noted concerns around identifying someone who is eligible for release to supervision and their actual release date within the 30-day window for XR-NTX administration. Also, because the re-entry period can be stressful and chaotic, revocation was common (and perhaps expected) if underlying SUD went untreated or inadequately treated. Therefore, identification of potential participants may need to expand to those serving a short sentence under discretionary detention (graduated sanction) or home incarceration.

Other concerns about meeting the needs of participants were raised in this focus group. One focus group participant noted, “Transportation is absolutely a problem around here,” a sentiment that was shared by the majority of the group. This statement highlighted expectations that providing ongoing medication administration at P&P rather than requiring a separate trip to a community clinic could significantly reduce barriers to retaining individuals on XR-NTX. DOC staff also emphasized the importance of participants in the pilot receiving counseling as part of their treatment with the clinic provider because they noted that a number of individuals on their caseloads had co-occurring mental health issues. Thus, staff strongly encouraged coordination of the XR-NTX administration appointment with counseling and case management sessions to coincide with the report day for study participants. DOC staff agreed they would need to maintain flexibility on reporting days in order to ensure that the clinic providers could come to the P&P office on these same days to provide care.

Finally, DOC staff mentioned stigma around MOUD in general and some misconceptions around XR-NTX specifically in the community and among professionals including jail staff. One respondent noted, “That negativity and stigma can go through the whole jail and can really affect whether people want to participate in this.” The group discussed the importance of providing education about XR-NTX for both the jail staff and medical personnel, in addition to individuals participating in the study. One respondent noted, “I think it would be helpful if we did some type of education for the staff there [at the jail], especially the medical because I’m sure they’re going to be probably fielding some general questions. If they don’t support it, I think that that could really cause people not to want to participate.” Overall, DOC staff felt like jail staff and medical providers would be receptive to additional XR-NTX education during study implementation.

A second focus group was completed with health care providers and staff at the partnering FQHC to understand better their standard of care, client needs, challenges of initiating and sustaining XR-NTX treatment, and ancillary case management and counseling services. Findings indicated that the clinic XR-NTX standard of care involved an assessment with a detailed medical and psychosocial history, urine drug screen, and laboratory bloodwork (metabolic panel, liver function tests, complete blood count). If patients do not have health insurance, staff work with patients to enroll them in Medicaid and obtain XR-NTX pre-authorization. All patients are provided with case management and are expected to participate in counseling sessions. Whenever possible, these appointments are all scheduled for the same day as the XR-NTX administration to reduce the burden of additional travel for patients. As long as patients are eligible (e.g., opioid negative urine sample, not in liver failure), clinic staff attempt to schedule patients the day after the medical evaluation for the oral naltrexone challenge (50 mg, 30-min wait period) and XR-NTX administration. At the time of the focus group, staff were only able to operate within their physical clinic location and 14 patients on XR-NTX was considered a “pretty big” clinic caseload.

In adapting their procedures for providing XR-NTX in the jail, FQHC staff stressed the importance of coordination with the jail administration to be able to provide all services (e.g., counseling, case management) in a private location. They felt strongly that maintaining confidentiality was critical in the jail setting and having the space to meet with a patient one-on-one was necessary. One of the identified challenges was that clinic staff did all of their charting electronically, which would not be feasible in the jail due to computer and internet restrictions. It was agreed that paper copies of forms could be collected for the jail medical visits and later entered into their electronic health record. Finally, another potential concern was the expectation that jail staff may negatively view using medications, which could be challenging for the study. With additional education and training, however, FQHC staff noted that views around medications can change, which also may be an important consideration for the study. One staff noted, “After I worked with a physician who prescribed Suboxone®, my view of Suboxone® is way different than it used to be.” Similar to the DOC staff focus groups, the importance of education around XR-NTX to reduce stigma was an important finding.

### Decision

Decisions related to initial DOC protocol adaptations were grounded in the stakeholder focus group findings, the empirical literature, ASAM (2015) guidelines for XR-NTX administration, and medication guidelines from the FDA package label (https://www.vivitrol.com/content/pdfs/medication-guide.pdf). Adaptations to the DOC protocol included more detailed procedures for screening, medical evaluation, and XR-NTX administration, as well as attention to culturally relevant language and examples to target rural Appalachian individuals with OUD (as shown in Table 1).

**Table 1:** Initial modifications to protocol based on focus groups

	DOC Protocol	XR-NTX Pilot Adaptations For Research Study
Patient identification	Participant in DOC Substance Abuse Program (SAP), modified therapeutic community during incarceration	Individuals in jail preparing for release; screening protocols to identify transition to supervision in target county and release dates (including court dates and pleas)
Patient eligibility	Only state inmates in state-funded jail and prison SAP programs with OUD who meet eligibility criteria	All individuals (regardless of classification) in one target rural county jail with an OUD transitioning to community supervision in the same county who meet eligibility criteria
OUD and physical dependence determination	Assessment of OUD through state data collection system, includes DSM criteria; negative urine drug screen	Additional tools were included in screening and assessment including DSM5 checklist assessment for OUD, negative opioid urine screen, and Clinical Opioid Withdrawal Scale (COWS) total score indicating no withdrawal
Oral naltrexone dosing, First XR-NTX administration and other ancillary services during incarceration	XR-NTX administered by corrections health care and any ancillary services provided through SAP (during incarceration) after taking 50 mg oral naltrexone once daily for 3 days	XR-NTX administered by one community clinic partner in the jail; session includes counseling and case management. XR-NTX injection given two hours after tolerating single dose of oral naltrexone 12.5 mg
On-going XR-NTX administrations in the community	Referrals made to community providers based on re-entry plan	Second XR-NTX administration scheduled at the clinic [comparison condition] or at the P&P office [experimental condition] (coordinated with report days) based on random assignment
Patient documentation	Medical evaluation results (including laboratory results) and XR-NTX administration notes included in individuals’ medical record at the facility	Medical evaluation results, XR-NTX administration notes, counseling and case management notes recorded on paper forms in the jail and transferred to electronic medical record upon return to the clinic

In addition, because of time and travel required to both the jail (for the clinical staff) and the P&P office following release (for the individual), protocol decisions included ensuring counseling and case management on the same day as the XR-NTX administration for individuals enrolled in the study.

### Administration

Overall, the walk through of the adapted protocol during the theater test indicated that the critical components of screening, assessment, medical evaluation, medication initiation, and continuity of care were consistent with the standard of care in addressing the volunteer’s needs. The theater test did reveal specific areas of training needs for the clinical team to adapt their traditional clinical practice to a clinical trial. For example, completion of each of the protocol forms to determine participant eligibility for the trial required more training on attention to detail and documentation in the jail (as compared to what was needed in the clinic environment). As a result, additional questions were added to the medical history and assessment to document OUD.

### Production and Topical Expert review

A more refined version of the adapted protocol was produced based on the theater test findings. Each section of the protocol was clearly delineated by sections (e.g., Screening and Recruitment, Medical Evaluation, Initial Injection, Community Injections) and included procedures for both research and clinical staff. Each section of the refined protocol included step-by-step procedures, as well as relevant research forms for documentation.

Reviews by both our study team and Topical Expert yielded additional suggestions during this stage of protocol refinement. One change included supporting the recommended change from the original protocol related to the oral naltrexone challenge of 50 mg for 3 days to 12.5 mg 2 h before the XR-NTX injection, which was also supported by empirical research on XR-NTX administration with correctional populations at re-entry (Friedmann et al., 2012; Gordon, Vocci, Fitzgerald, O’Grady, & O’Brien, 2017; Lee, Friedmann, et al., 2015; Lincoln, Johnson, McCarthy, & Alexander, 2018). This change also required commitment from the FQHC clinical team to change their standard of care protocol from a single 50 mg dose followed by observation to ensure no precipitated withdrawal for 30 min to a single 12.5 mg dose with 2-h observation period for research study participants only, which was supported as the 2-h wait period was an ideal time to deliver counseling and initiate case management services prior to jail release.

Other important findings from this phase included clarity on the timeframe for community XR-NTX administration if an individual passed the target window for the second administration following jail release (~ 30 days). The goal was to retain patients on XR-NTX if the participant desired it and it remained medically safe to continue. Thus, in the case of suspected or confirmed lapse/relapse, the protocol required a negative opioid urine drug screen, no recent self-report of opioid use, 12.5 mg oral naltrexone challenge tolerance (if needed as determined by clinical judgement), and a clinical opioid withdrawal scale (COWS) score < 8 prior to considering the next XR-NTX administration.

### Integration

Feedback from the Topical Expert and study team was then integrated into the final draft of the adapted XR-NTX protocol. The PI, physician Co-I, and study coordinator met with key stakeholders from the partnering jail and P&P office, as well as the FQHC clinical team to walk through the protocol to assess feasibility. At this meeting, final decisions were made about logistics (e.g., which rooms to use, channels of communication, etc.) in both the jail and the P&P office. One final review of the completed protocol was conducted by the research team prior to launching training for the pilot trial.

### Training

The Training phase included education for both research staff and the FQHC team. One full-day interactive training session was conducted with research staff on the adapted XR-NTX protocol. Training material was both didactic (e.g., overview of key content by the PI) and interactive (e.g., role play with volunteers as practice sessions) and primarily focused on confidentiality and informed consent, recruitment and screening procedures, and data collection procedures at baseline and in the community at 3 months post-release from jail. A separate one-day training was conducted with the FQHC health care team including the nurse practitioner, registered nurse, therapist, case manager, medical records staff, and clinic administrators. The training was led by the physician Co-I and included a didactic presentation on each section of the adapted protocol with specific focus on procedures in the medical evaluation and XR-NTX administration sessions. Training for health care providers also included a focus on research documentation, medication shipment and storage, and maintaining data records. All research and clinical team members were included on the IRB protocol and completed required protection of human subjects and NIH Good Clinical Practice trainings. The study physician Co-I, who also serves as the Medical Monitor, provided one additional half-day training on assessing, documenting, and reporting adverse events. Finally, in preparation for the pilot trial launch, a two-hour training was also provided for officers in the partnering P&P office in order to raise awareness about the study, discuss XR-NTX administration, provide details about the adapted XR-NTX protocol and logistics, and answer any questions.

## Discussion

Justice-involved individuals with a history of OUD are among the most vulnerable because they often experience sustained abstinence with subsequent loss of opioid tolerance during incarceration and, without linkage to treatment, they are at high risk for overdose during community re-entry. This study is grounded in the empirical evidence for XR-NTX for re-entering offenders, but expands the work to examine an innovative model to support continuity of care with XR-NTX after jail release by co-locating MOUD treatment within the P&P office. This paper describes the first seven steps of the ADAPT-ITT framework for the research-based adaptation of the DOC protocol for XR-NTX administration in substance use treatment programs to a non-traditional, non-treatment, re-entry model of care (jail and P&P office) in a rural community. This is the first study to utilize the ADAPT-ITT framework for a medication administration protocol.

Findings from stakeholder focus groups, study team review, topical expert review, and a theater test suggested that there were critical adaptations needed at the patient and clinic level. In the original DOC protocol, potential patients are identified through existing SAP programs within prisons, jails, and community custody settings. While the number of treatment slots have grown in recent years in the state, DOC currently has the capacity to treat about a quarter of inmates with a history of substance use (CJKTOS, 2019). Thus, to implement the adapted XR-NTX protocol in a jail without a SAP program required specific adaptations for patient identification and eligibility not currently included in DOC protocol. Other critical adaptations included identifying a target release date in order to administer XR-NTX while incarcerated and also making sure individuals were within a target window for release to the community. The pilot trial test of the adapted protocol requires a flexible target window in order to accommodate administering XR-NTX prior to release for both state inmates (with set parole dates to the community) and county inmates (usually people in jail with less predictable release dates who are then placed on probation). Finally, because individuals approached about the pilot study may be hearing about XR-NTX for the first time, the screening procedures and medical evaluation included education about XR-NTX benefits and potential side effects.

The DOC protocol for the administration of XR-NTX included counseling provided through the state SAP programs and case management provided through a social service clinician in the P&P office following release. Because the adapted protocol will be implemented in a non-treatment jail setting, critical modifications were needed by the community partner in order to provide counseling and case management during the same session as XR-NTX initial and follow-up administration in order to reduce additional travel burden for clinic staff and volunteers. In practice, once a participant is cleared for XR-NTX induction following the medical evaluation, this includes a therapist and case manager accompanying the nurse to the jail. Following administration of the oral challenge of naltrexone, the therapist and case manager meet with the participant prior to receiving XR-NTX. Working with one community provider will also allow continuity of care from the jail (medical evaluation and XR-NTX induction) to the community (on-going medication administrations post-release always coupled with counseling and case management at the medication administration visit).

### Lessons learned

DOC protocol adaptations were primarily related to context and content. Initiation of XR-NTX is guided by federal and state guidelines in best practices which include screening for OUD, a medical evaluation with laboratory tests (urine toxicology, metabolic panel, liver function tests, complete blood count, pregnancy testing), and lack of opioid physical dependence (SAMHSA, 2020). Each of these core components were maintained in the adapted protocol. The primary differences are noted in patient recruitment, medication induction doses, urine drug screening requirements (became more lenient allowing for non-opioid tests to be positive) and the continuity of care from jail to the community.

With regard to patient recruitment, it is important to recognize the stigma around MOUD in rural areas may affect participation. It was noted in both stakeholder focus groups that stigma around non-medical opioid use in general, as well as MOUD treatment (and XR-NTX specifically), is very common in this rural area – including among a number of professional organizations (including criminal justice staff). This has been consistently noted in the literature for criminal justice organizations, particularly P&P and jails (e.g., Bunting, Oser, Staton, Eddens, & Knudsen, 2018; Friedmann et al., 2012). Being mindful of the potential stigma around medications during the recruitment and community retention is important, as well as increasing education for participants and stakeholders on the benefits of medications.

DOC focus group findings also suggested that re-entry to the community from jail is a high-risk and chaotic period for justice-involved individuals. It is possible that life events – such as finding safe housing or stable employment – may supersede the perception of the importance of treatment and negatively impact adherence to XR-NTX post-release, which has been noted elsewhere (Velasquez et al., 2019). In addition, if an individual relapses or engages in criminal activity after release, he/she may abscond from supervision – making XR-NTX administration in the community supervision office not feasible. These issues of feasibility and acceptability are critical to monitor during the pilot trial test in this non-traditional, non-clinical environment in preparation for a larger clinical trial.

Partnering with a local FQHC as the health care provider for the study is a tremendous asset, particularly in ensuring familiarity with the clinical protocol for XR-NTX administration. A main advantage is the potential to enhance the continuity of care from the medical evaluation and initial XR-NTX administration in the jail to ongoing XR-NTX administrations and supported services in the community. Challenges noted during the planning process included participation in the research trial as possibly burdensome for clinic staff considering their standard clinical responsibilities. Specifically, in addition to patient care (nurses) and therapeutic caseloads (therapist and case manager), FQHC clinic team members had to travel to the jail for initial visits with the participants, as well as complete additional forms for OUD assessment and documentation, medical histories, and study protocol adherence checklists. While this required additional training by the research team, having a shared vision for the success of the pilot trial was the foundation for ensuring resource allocation (e.g., supporting clinic staff salary) to best balance the research trial in the context of day-to-day clinical operations. Clinical staff also received additional professional development training on managing clinical trials, which was useful for their future work. In addition, balancing the workload with the research staff so that they assist with screening procedures and monitoring participant forms and documentation to assist with potential burden on the clinical team was also beneficial.

Along these lines, it is also important to note that most clinical trials take place in laboratory or clinical research environments. Small community FQHCs and criminal justice settings, such as jails and community supervision offices, are less “controlled” from a research perspective and may be more vulnerable to policy level changes that affect study enrollment. Examples include closing the jail and/or P&P offices due to financial constraints, reorganizations, or public health crises (e.g., COVID-19 pandemic). In addition, in controlled laboratory trials, study treatment cost is often covered by the project, while in real-life settings, it can be more heavily impacted by health plan coverage (which may limit treatment access).

### Limitations

The primary limitation of this descriptive level qualitative research is the lack of generalizability beyond the stakeholders of two professional groups (corrections and health care staff) in one small rural community in Appalachia. While recognizing this limitation, focus group feedback was used primarily for the purpose of intervention adaptation in this study rather than a broader focus on qualitative assessment. Our reliance on a systematic framework to guide the adaptation process enhances the rigor of study qualitative methods. The current study is targeted to administration of XR-NTX, which limits generalizability to other MOUDs including buprenorphine and methadone. However, the use of the ADAPT-ITT framework provides a systematic and iterative process that could be replicated in other settings, for other evidence-based medications such as buprenorphine, and for other target populations. Future research should also include justice-involved individuals during the planning and adaptation process, a potential limitation in the current study and a notable gap in the broader literature (Puglisi, Bedell, Steiner, & Wang, 2019).

## Conclusion and next steps

Despite these limitations, the significance of this study for health and justice officials lies in the response to the opioid crisis in rural Appalachia, the increased vulnerability of rural individuals with OUD, and the dearth of available and accessible evidence-based treatment which can be addressed through an innovative partnership between a jail, local P&P, and a clinic. This paper overviews study activities and findings through the first seven phases of the ADAPT-ITT framework. Next, we will complete the final ADAPT-ITT phase, *Testing,* which involves launching the pilot randomized clinical trial (RCT) to screen, assess, and medically evaluate eligibility for XR-NTX. The pilot RCT will also examine the continuation of XR-NTX from induction in the jail to on-going community administrations in one of two test sites following jail release. The pilot RCT will inform whether co-locating treatment in real-world criminal justice settings, like P&P offices [experimental arm], can successfully reduce barriers to on-going community treatment (such as transportation), while also signifying support for treatment engagement from the criminal justice system compared to on-going treatment at the clinic [comparison condition]. Findings from the pilot trial have the potential to contribute to the OUD treatment field by advancing knowledge on innovative service delivery models to reduce high-risk opioid use and related health disparities among hard-to-reach, underserved justice-involved populations.

## Data Availability

The datasets used and/or analyzed during the current study are available from the corresponding author on reasonable request.
